# Use, adoption, and effectiveness of tippy-tap handwashing station in promoting hand hygiene practices in resource-limited settings: a systematic review

**DOI:** 10.1186/s12889-020-09101-w

**Published:** 2020-06-26

**Authors:** Balwani Chingatichifwe Mbakaya, Fatch Welcome Kalembo, Maggie Zgambo

**Affiliations:** 1 St John’s Institute for Health, P.O. Box 18, Mzuzu, Malawi; 2 Mzuzu University, Private Bag 201, Luwinga, Mzuzu 2, Malawi

**Keywords:** Tippy-tap, Handwashing station, Adoption, Effectiveness, Hand hygiene practice

## Abstract

**Background:**

Tippy-taps are locally made devices for washing hands with running water. They are simple and low-cost, enabling technology that provides adequate water sources, handwashing stations and motivation for people to prioritise handwashing. This systematic review aimed to establish the use, benefits, adoption and effectiveness of enabling technology; tippy-tap handwashing station, in resource-limited settings.

**Methods:**

We systematically searched for articles in the PubMed, EMBASE, PsycINFO, AMED, CINAHL, DOAJ and Google Scholar databases guided by the acceptable best practice developed by the PROSPERO and COCHRANE for systematic search and selection of articles. Search terms such as tippy-taps, enabling technology, hand-washing station, hand-washing behaviour, diarrhoea, respiratory infection, increase handwashing behaviour were used. In addition, a PRISMA flow diagram was used to elaborate on the number of articles retrieved, retained, excluded and reasons for every action. Studies that used tippy-tap hand washing station as a handwashing facility regardless of the design were included in this review. A mixed method appraisal tool was used to appraise studies.

**Results:**

Twenty articles met the eligibility criteria. The use of tippy-taps for handwashing by household members or school children was reported by authors of 16 studies, and it ranged from 2.7 to 80%. The availability of tippy-taps increased handwashing and use of soap among participants. Furthermore, the majority of people who were oriented to tippy-taps or recruited to tippy-tap studies built their tippy-tap stations even after the promotional activities or programs had ended. In one study, tippy-taps were reported by participant to be effective in preventing episodes of stomach pain among participants.

**Conclusion:**

Tippy-tap handwashing station could help in promoting handwashing practice in resource constraint settings. Future studies are needed to evaluate the effectiveness of tippy-tap hand washing station on preventing water and hygiene-related infections.

## Background

The United Nations International Children’s Emergency Fund (UNICEF) estimate that 884 million people in the world lack access to basic drinking water supply services [1]. The majority of these people live in rural areas of low and middle-income countries [1]. Lack of improved water sources in these areas is problematic not only to the households but also to the public facilities such as hospitals and schools [2]. The World Health Organisation (WHO) states that 38% of healthcare facilities lack an improved water source, 19% lack improved sanitation, and 35% lack water and soap for handwashing in developing countries [2]. In addition, more than half of all primary schools in developing countries do not have adequate water facilities and nearly two-thirds lack adequate sanitation [1]. Where water or water stations are not readily available, neglecting hand washing is not uncommon. Failure to wash hands after visiting the toilet, before eating or feeding a child, before and after preparing food, and after changing and cleaning up a child who has used a toilet, increases the risk of contracting or spreading diarrheal and respiratory-related diseases [2, 3]). The inadequacy of water supply, sanitation and hygiene cause the death of a child every minute, 80% of childhood diseases, 272 million days of school absenteeism and other health conditions such as diarrhoea and respiratory disorders in the general population [4, 5].

**Fig. 1:**
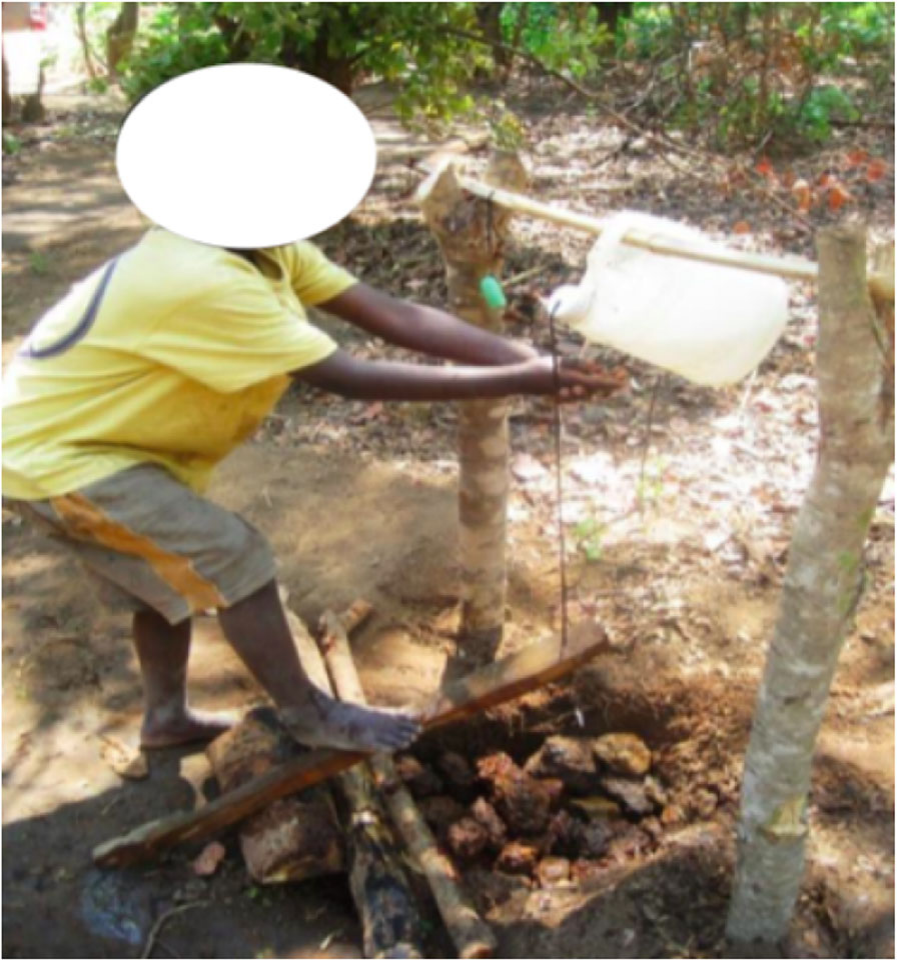
A boy washing hands using tippy-tap. Source: UNICEF/Zambia/2012/Asindua [8]

**Fig. 2:**
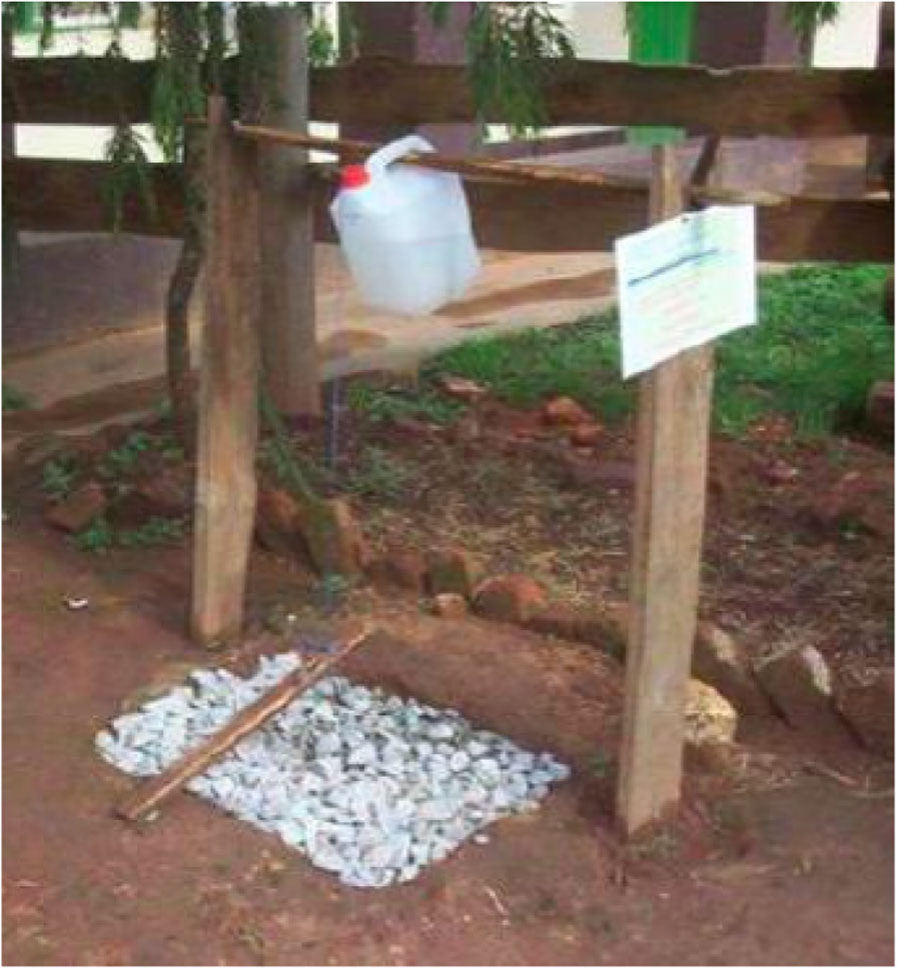
An examples of a tippy-tap. Source: Mark Tiele Westra [9]

Although lack of resources and modern technology are commonly associated with the inadequate handwashing stations, low cost and simple handwashing and technology such as tippy-taps may provide adequate water sources, stations and motivation for people to prioritise handwashing [6]. Tippy-taps are simple and economic handwashing stations, made with locally available materials including plastic containers, jerry cans or gourds, and do not depend on a piped water supply [6]. Biran [7] describes a tippy-tap as ‘*a device consisting of a small (three or five-litre) jerry can be filled with water and suspended from a wooden frame. A string is attached to the neck of the jerry can that can be tied to a piece of wood at ground level. Pressing on this piece of wood with the foot, tips the jerry can to release a stream of water through a small hole. Soap is suspended from the frame beside the jerry can*’ (See Figs. 1 and 2). Furthermore, tippy-taps are easy to construct, use very little water, easier to use and only soap is touched, thereby making handwashing very hygienic because it avoids contamination of the jerry can, unlike the real tap [7]. Tippy-taps could be a technology of choice for reducing diarrheal and respiratory disorders and deaths that are associated with lack of water, inadequate handwashing stations and practices through controlling factors that hinder handwashing practice such as unavailability of handwashing station, water and soap [10]. Following this, it should, therefore, be noted that reducing infectious diseases that occur due to unhygienic hand practices takes more that handwashing education, the handwashing stations, water and soap equally play a major role in reducing.

The first tippy-tap was constructed by Dr. Jim Watt and Jackson Masawi of the Salvation Army in Chiweshe, Zimbabwe, and was called the Mukombe in the 1980s. The Mukombe is a type of gourd or calabash, which is used as the can [11]. Since then, many different versions of tippy-taps have emerged in different parts of the world, depending on the accessibility and types of available local materials. Tippy-taps, although simply constructed from locally affordable and accessible materials, could be the suitable handwashing stations for underdeveloped settings that often lack adequate water for handwashing. The average amount of water used for handwashing using tippy-taps is far much less compared to ordinary handwashing stations such as taps. Comparatively, a good hand wash using tippy-tap could use only 50 mls of water, while washing hands using tap water may utilise up to 500 mls of water [11]. Furthermore, tippy-taps could help to increase handwashing behaviour in schools because it is appealing to children since it is humorous and easy to use, consequently cutting the number of deaths in children that occur due to health conditions associated with hand hygiene practices [7]. Enabling technology is one of the factors that externally influence individual’s probability to accomplish a behaviour [7]. The UNICEF and WaterAid recommend the use of tippy-taps in schools and family houses next to the latrines [12, 13]. Tippy-tap is possibly the best known low cost enabling technology for handwashing [7] and currently, tippy-taps are commonly used in East and Southern Africa in countries like Uganda, Rwanda, and Zambia [13].

The aim of this systematic review, therefore, was to gather, consolidate and quantify the evidence of the use, benefits, adoption and effectiveness of tippy-tap handwashing station in promoting hand hygiene practices in resource-limited settings. Promotion of handwashing behaviour was the main outcome in this systematic review. The secondary outcomes were use, adoption, benefits and effectiveness of tippy-taps. The questions that were addressed by this review are: 1) How does the use of tippy-tap handwashing stations promote hand hygiene practices in a resource-limited setting? 2) How effective are tippy-taps in promoting hand hygiene and reducing water and hygiene-related infections?

## Methods

### Protocol

This review was guided by the acceptable best practice developed by the PROSPERO and COCHRANE for systematic search and selection of articles. The protocol was published in the PROSPERO database with registration number CRD42017074331 [14].

### Inclusion criteria

All studies that used tippy-tap handwashing station as a handwashing facility regardless of the design were included in this systematic review.

### Exclusion criteria

Papers written in languages other than English and articles with studies conducted in developed countries were excluded.

### Information source /search strategy

The following database sources were used to gather the required information; Medline, EMBASE, PsycINFO, AMED, CINAHL, DOAJ and Google Scholar. MeSH terms such as hand hygiene, hand disinfection, hand washing, handwashing, hand washings, washings, hand scrubbing, scrubbing, infection, cross-infection, waterborne, waterborne disease, water related diseases, water diseases and diarrhoea were used during searching for the articles to ensure accuracy. Besides MeSH terms, keywords were also combined using Boolean operators OR and AND. The following key terms and MeSH terms were used: Tippy-taps, OR Enabling technology OR Hand-washing station OR Hand washing interventions OR Hand washing strategies OR Hand washing programs AND Hand wash OR Hand washing OR Hand washings OR Handwashing OR Hand washing behaviour, OR Hand washing techniques OR Hand hygiene OR Hand disinfection OR Hand or Washings OR Hand scrubbing AND Use OR Usefulness OR Utilisation OR Benefit OR Advantages OR Effectiveness OR evaluation AND Promotion OR Sustainability OR Adoption OR Appropriateness AND Prevention OR Control OR Limit AND diarrhoea, OR dysentery OR waterborne disease OR bloody stool OR Loose stool OR Respiratory Infection OR Infection OR Cross infection (see Table 1). Keywords were also used to search for articles in Google Scholar. Efforts were made to identify both published and unpublished interventional studies by manually checking the reference list of the articles that met the inclusion criteria. Several strategies were used to identify unpublished studies. First, we reviewed the methodology and reference list of the included studies to assess if they identified any unpublished research related to the review question. Second, we manually searched conference proceedings such as Development International Conference, Water Engineering and Development Centre and the University of North Caroline Water and Health Conference for any suitable studies. Further searches were conducted in clinical trial website such as ClinicalTrials.gov website (https://clinicaltrials.gov/). Efforts were also made to contact the authors of the unpublished studies. Reference lists of the included studies were checked and hand searching in the key journals was also done. The search period for the research articles in the mentioned databases was from the inception of the databases to July 2019. The search for the eligible studies in the database was conducted between September 2017 to July 2019.

**Table 1:** Search strategy

Databases	Search	Search words/terms	Results
CINAHL	Title & abstract	Tippy-taps, OR Enabling technology OR Hand-washing station OR Hand washing interventions OR Hand washing strategies OR Hand washing programs AND Hand wash OR Hand washing OR Hand washings OR Handwashing OR Hand washing behaviour, OR Hand washing techniques OR Hand hygiene OR Hand disinfection OR Hand or Washings OR Hand scrubbing AND Use OR Usefulness OR Utilisation OR Benefit OR Advantages OR Effectiveness OR evaluation AND Promotion OR Sustainability Or Adoption OR Appropriateness AND Prevention OR Control OR Limit AND diarrhoea, OR dysentery OR waterborne disease OR bloody stool OR Loose stool OR Respiratory Infection OR Infection OR Cross infection	4
MEDLINE	Title & abstract	Tippy-taps, OR Enabling technology OR Hand-washing station OR Hand washing interventions OR Hand washing strategies OR Hand washing programs AND Hand wash OR Hand washing OR Hand washings OR Handwashing OR Hand washing behaviour, OR Hand washing techniques OR Hand hygiene OR Hand disinfection OR Hand or Washings OR Hand scrubbing AND Use OR Usefulness OR Utilisation OR Benefit OR Advantages OR Effectiveness OR evaluation AND Promotion OR Sustainability Or Adoption OR Appropriateness AND Prevention OR Control OR Limit AND diarrhoea, OR dysentery OR waterborne disease OR bloody stool OR Loose stool OR Respiratory Infection OR Infection OR Cross infection	7
AMED	Title & abstract	Tippy-taps, OR Enabling technology OR Hand-washing station OR Hand washing interventions OR Hand washing strategies OR Hand washing programs AND Hand wash OR Hand washing OR Hand washings OR Handwashing OR Hand washing behaviour, OR Hand washing techniques OR Hand hygiene OR Hand disinfection OR Hand or Washings OR Hand scrubbing AND Use OR Usefulness OR Utilisation OR Benefit OR Advantages OR Effectiveness OR evaluation AND Promotion OR Sustainability Or Adoption OR Appropriateness AND Prevention OR Control OR Limit AND diarrhoea, OR dysentery OR waterborne disease OR bloody stool OR Loose stool OR Respiratory Infection OR Infection OR Cross infection	14
PsychINFO	Title, abstract & full article	Tippy-taps, OR Enabling technology OR Hand-washing station OR Hand washing interventions OR Hand washing strategies OR Hand washing programs AND Hand wash OR Hand washing OR Hand washings OR Handwashing OR Hand washing behaviour, OR Hand washing techniques OR Hand hygiene OR Hand disinfection OR Hand or Washings OR Hand scrubbing AND Use OR Usefulness OR Utilisation OR Benefit OR Advantages OR Effectiveness OR evaluation AND Promotion OR Sustainability Or Adoption OR Appropriateness AND Prevention OR Control OR Limit AND diarrhoea, OR dysentery OR waterborne disease OR bloody stool OR Loose stool OR Respiratory Infection OR Infection OR Cross infection	3
DOAJ	Title, abstract & full article	Tippy-taps, OR Enabling technology OR Hand-washing station OR Hand washing interventions OR Hand washing strategies OR Hand washing programs AND Hand wash OR Hand washing OR Hand washings OR Handwashing OR Hand washing behaviour, OR Hand washing techniques OR Hand hygiene OR Hand disinfection OR Hand or Washings OR Hand scrubbing AND Use OR Usefulness OR Utilisation OR Benefit OR Advantages OR Effectiveness OR evaluation AND Promotion OR Sustainability Or Adoption OR Appropriateness AND Prevention OR Control OR Limit AND diarrhoea, OR dysentery OR waterborne disease OR bloody stool OR Loose stool OR Respiratory Infection OR Infection OR Cross infection	8
Google Scholar	Title & abstract	Tippy-taps and handwashing	4040
EMBASE	Title, abstract & full article	Tippy-taps, OR Enabling technology OR Hand-washing station OR Hand washing interventions OR Hand washing strategies OR Hand washing programs AND Hand wash OR Hand washing OR Hand washings OR Handwashing OR Hand washing behaviour, OR Hand washing techniques OR Hand hygiene OR Hand disinfection OR Hand or Washings OR Hand scrubbing AND Use OR Usefulness OR Utilisation OR Benefit OR Advantages OR Effectiveness OR evaluation AND Promotion OR Sustainability Or Adoption OR Appropriateness AND Prevention OR Control OR Limit AND diarrhoea, OR dysentery OR waterborne disease OR bloody stool OR Loose stool OR Respiratory Infection OR Infection OR Cross infection	12
Reference search from other sources	Title, abstract & full article	Tippy-taps, OR Enabling technology OR Hand-washing station OR Hand washing interventions OR Hand washing strategies OR Hand washing programs AND Hand wash OR Hand washing OR Hand washings OR Handwashing OR Hand washing behaviour, OR Hand washing techniques OR Hand hygiene OR Hand disinfection OR Hand or Washings OR Hand scrubbing AND Use OR Usefulness OR Utilisation OR Benefit OR Advantages OR Effectiveness OR evaluation AND Promotion OR Sustainability Or Adoption OR Appropriateness AND Prevention OR Control OR Limit AND diarrhoea, OR dysentery OR waterborne disease OR bloody stool OR Loose stool OR Respiratory Infection OR Infection OR Cross infection	3
**Total records searched**	**4091**		
**Total articles** **included**	**20**		

### Study selection

Identified titles from the databases were extracted and imported to Endnote X7 Reference Management System. Thereafter, duplicates were removed. The abstracts of the retained titles were retrieved and manually assessed for potential eligibility. Full articles were retrieved for the retained abstracts and these were thoroughly assessed manually for eligibility. Assessing eligibility for the articles was done independently by two reviewers using the predefined inclusion and exclusion criteria. Any disagreement between the two reviewers over the eligibility of particular studies were resolved through discussion with a third reviewer.

### Data collection process

**Fig. 3:**
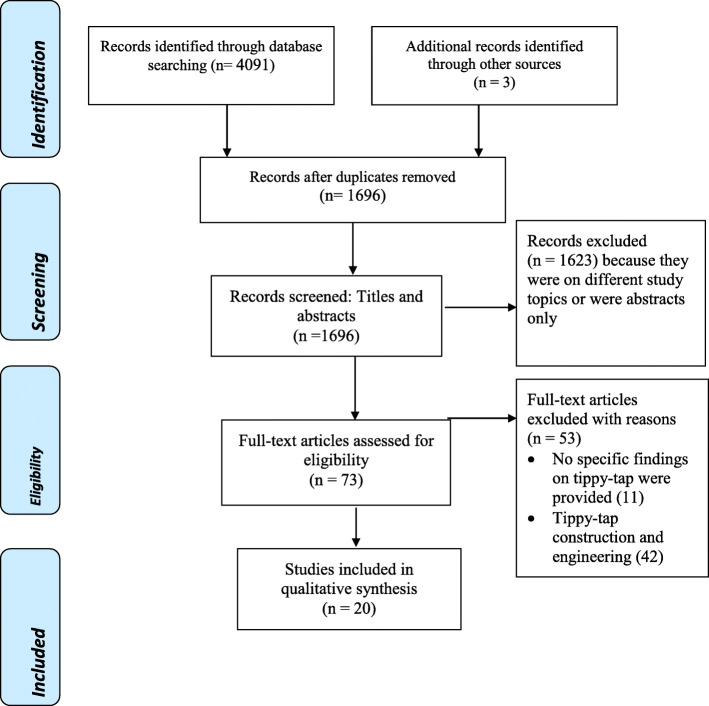
PRISMA Flow Diagram [15]

The process of data extraction started with database search of relevant articles using search terms while following the Preferred Reporting Items for Systematic Reviews and Meta-Analyses (PRISMA) [15] guidelines (see Fig. 3). A standardised form was used to extract data from the included studies for assessment of the study quality and evidence synthesis. The details included: author, year of study, type of participants, age, setting, country, sample size, study design, and methods, study purpose and objectives, intervention description, study outcome measures (see Supplementary material A). All relevant information was extracted from each article, summarised and documented (see Table 2). Two reviewers extracted data independently; discrepancies were identified and resolved through discussion with a third author. Missing data were requested from the corresponding authors of the study.

**Table 2:** Summary of studies

Author & year	Population				Sample size	Study design & methods		Objectives/aims	
	Participants	Age	Setting	Country			Study purpose/ Objective	Outcomes	Results
Abass (2018) [16]	Women	Adults (Age not provided)	Community	Nigeria	500 women in 10 communities	Qualitative and quantitative cross sectional survey. Data was collected through a questionnaire and observational checklist.	To improve livelihoods and promote safe sanitation, water and healthy living at home and community	Use and benefits of tippy-taps	Women who constructed tippy-taps at their place of business experienced a higher patronage of customers, which led to more sales. The customers were attracted to the business site because of safe sanitation and effective hand washing practices.
Aiemjoy et al., 2017 [17]	Children Heads of the Households	0–5 years Adults (age not provided)	Community	Ethiopia	255 children	Interviews, observations	To describe the prevalence of soil-transmitted helminths and intestinal protozoa in preschool children 0–5 years of age in seven communities in the Amhara region of Ethiopia, and to investigate associations between infection, household water and sanitation characteristics, and child growth	Use and adoption of tippy-taps	Tippy-tap was observed in approximately one in five households Soap was observed in over three-quarters of households
Biran (2011) [7]	Household heads	Adults Villagers (Age not provided)	Community	Uganda	44 interviews	Qualitative case study: semi-structured interviews. Data were collected through nine key informant interviews, forty-seven interviews with householders from model and non-model villages, and twenty-two spot-check observations of handwashing facilities.	To learn about the promotion of specific handwashing enabling technology (the tippy-tap) through a particular approach (the use of visiting health workers and village-level volunteers to provide health education and carry out household inspections in model villages) in Uganda	Promotion of hand hygiene practices (Increased hand washing) Benefits and adoption of a tippy-taps	The tippy-taps probably increased handwashing after latrine use by providing convenient soap and water, and by acting as a salient cue to wash hands. The tippy-taps were also attractive and easy for children to use and helped to foster the habit of handwashing among children. Tippy-taps were common, though not universal in model villages. Awareness of the tippy-taps did not necessarily translate into immediate action to obtain one. Tippy-taps were acceptable to householders and were thought to have many advantages compared to using a jerry can. Dissemination of information about the tippy-tap between villages and even between households within villages was limited. While quantitative data on handwashing rates were not collected, households with tippy-taps believed that their post-latrine handwashing rates had increased as a result of the taps. Respondents in non-model villages were largely unaware of tippy-taps. Not all tippy-taps had water in them- might be that they were not in use or had run out of water Researchers believed that participants adopted the tippy-tap technology because they were told to do so. One participant said she constructed tippy-tap because she had seen one, which looked modern and she wanted visitors to use it. Other participants built tippy-taps because they knew they would be visited by health assistants. Some participants constructed tippy-taps because of the campaign and feared fines. Participants were able to articulate genuine advantages of tippy-taps such as prevention of contamination and use of less water, but it was not clear whether this was just a repetition of the health message by health assistants or genuine motivation to build tippy-taps. One participant suggested that tippy-taps did not look attractive. Elderly participants said tippy-taps looked childish and unnecessary, people used to live longer before tippy-taps. Some participants complained that they needed to replace some parts yearly. Some participants had never heard about tippy-taps. No data to quantify handwashing changes.
Bresee et al. (2016) [18]	Children 39 female guardians	8–12 years And adults over 18 years	Schools and households	Zambia	5 schools were purposively selected. Teachers helped to purposively select students to participate in the study	Qualitative methods (Used open ended questions to ask guardians and pupils during focus group discussions) Two focus groups discussion (FDGs) for pupils at each school during phase I & 2 with the same pupils and guardians	To assess the potential for children to be change agents in five schools in rural Zambia.	Adoption of tippy-taps	Pupils engaged parents and siblings in constructing tippy-taps in their homes despite some parents indicating that they did not know what it was.
Cantrell, 2013 [19]	Households members	37.7 years average age	Community	Haiti	Household (*N* = 1198) and a latrine (*N* = 167) 26 communities 182 households	Survey that recorded household use of laundry pads, bath houses, hand-pumped drilled wells, health and hygiene education sessions, and latrines as well as demographic data	To examine and describe potential strengths, weaknesses, and opportunities within the intervention program as well provide recommendations for future WASH projects in Haiti and in other developing countries.	Use of tippy-taps	Use of tippy-taps in many communities ranged from 0 to 40%.
Chisanga 2018 [20]	Mother and caregivers of 0–23 months olds	Adults (Age not provided)	Community	Tanzania	161 mothers and caregivers	Questionnaire Interviews and FGDs	To assess the sustainability practices of Mwanzo Bora Nutrition Program at Kilolo district	Use and adoption of tippy-taps	80% of the participants had tippy taps. 80% of those who had tippy-taps were using them before and after using toilet (*p* ≤ 0.05). 55% reported that they were still using tippy-taps after the implementation of the program (*p* ≤ 0.05).
Chiziwisano et al., 2019 [21]	Households with a child aged between 3 and 24 months	Adults (Age not provided)	Community and Households	Malawi	21 households 323 participants	Mixed methods. Data collected through household survey Household surveys (*n* = 323), checklists (*n* = 31), structured observations (*n* = 80), and microbiological food samples (*n* = 20)	(1) To identify practices and associated factors at household level related to food contamination, child mouthing, handwashing practices and kitchen utensils.	Use of tippy-taps	A specific place for handwashing, mostly tippy taps was found in 51% of households. Only 19% of handwashing facilities had soap and water. The majority (64%) of handwashing facilities were located near the latrine.
Christensen et al. (2015) [22]	Caregivers of 4- to 16-month-old children in the first study area and pregnant women in their second or third trimester and caregivers of children under 3 months of age in the second study area	Adults (Age not provided)	Households in 72 villages	Western Kenya	499 subjects	Pilot cluster randomized trial	The study’s scientific objectives are to (1) determine if WASH interventions aid in early child development, (2) determine if the combination of WASH interventions is more beneficial than a single intervention alone, and (3) determine if the com- bination of WASH interventions plus nutrient supplements is more beneficial than any of the interventions or supplements alone.	Use of tippy-taps	Enumerator-observed indicators of use of tippy taps (availability of both soap and water) ranged between 72 and 85%.
Contzen et al., 2015 [23]	Primary caregivers of households	Adults (Age not provided)	Community	Ethiopia	462	Quasi-experiment with pre-post design and four arms An intervention was administered in four arms of the study. In arm 1, the control group, only education was implemented; arm 2 received education plus public commitment; arm 3 received education plus tippy-tap promotion; and arm 4 received education, public commitment and tippy-tap promotion	To test the hypothesis that evidence- and theory-based interventions, especially when matched to the target population’s needs, are expected to perform better than common practice.	Use and adoption of tippy-taps	In kebeles 3 and 4, nearly 100% of the households followed the promotion and invested material and time to construct for themselves a tippy- tap. Three months after termination of the intervention, tippy-taps were in use with water and soap being present in up to 83% of the households (kebele 4). Pre-post data analysis on self-reported handwashing revealed that the population-tailored interventions, and especially the tippy-tap-promotion, performed better than the standard education intervention. 94 to 99% of participants in the intervention arm built the tippy-taps and recognised tippy-tap as their designated place for handwashing.
Dajaan et al. (2018) [24]	Children and Headmasters	Children and adults (Age not provided)	Schools	Ghana	300 children and 10 headmasters in 10 selected schools.	A cross sectional Data were collected using questionnaires and observation checklist regarding socio-demographic characteristics, knowledge of hand washing, hand washing practices and availability of hand washing facilities in the selected schools.	To determine the availability of hand washing facilities, hand washing knowledge and practices among public primary schools in Kintampo Municipality.	Promotion of hand hygiene practices (Increased hand washing) Use of tippy-taps	37.67% of participants washed their hands in order to prevent diseases, 53.33% had never been educated on how to wash their hands, 23.33% of the children demonstrated correctly on how to wash hands, over 15% washed -lean running water, while 23.33% wipe their hands using handkerchiefs. 40% indicated that it is necessary to wash hands after visiting toilet. 42.33% cited lack of water as the barrier to hand washing. 39.88% always washed their hands with soap after using the toilet. 60% of the schools had hand washing points. 30% of the schools had clean running water. 20% had one or more tippy-taps.
Hurtado (1994) [25]	Mothers with children under three years of age who had water taps and latrines in their homes	Children (Age not provided)	Community	India	300 mothers, 40 indepth interviews	Qualitative	To obtain in-depth information on beliefs, perceptions, and motivation with regard to water, and the behaviours related to the handling and use of water	Use and benefits of tippy-taps	The perceived benefit of tippy-tap use was that it uses less water and soap than the usual method of hand washing because the soap is not placed where it gets wet and soggy but hangs up and dries. It was not easy to wash hands of very young children with the tippy-tap. Another potential problem was that older children may play with the device, thus destroying it or wasting water. Although mothers did not mention it, it is recognized that the device requires extra water, time, and work to install, use, and maintain.
Kamuteera et al. 2018 [26]	Stakeholders, including: (i) households; (ii) institutions such as schools, churches, and police and prison barracks; and (iii) local government officials and non-governmental organisations (NGOs).	Adults Stakeholders (Age not provided)	Community	Uganda	138 protected springs and ten gravity-flow schemes (GFSs) were surveyed. Household-level data were collected from 150 households, and four NGOs provided insight into their experiences and practices.	Survey Data collection occurred through direct visual observation, onsite dialogue with individuals and groups, telephone and electronic correspondence with stakeholders, and structured questionnaires. Three types of questionnaires were developed to cater to various stakeholders, including: (i) households; (ii) institutions such as schools, churches, and police and prison barracks; and (iii) local government officials and NGOs.	To assess the roles that training and monitoring have played in WASH projects in Rukungiri District. To examine the theoretical possibility of selling nutrients recovered from sanitation to support the ongoing monitoring and operating needs of local water systems	Promotion of hand hygiene practices (Increased hand washing) Use of tippy-taps	Most households had dish drying racks, but hand-washing facilities were extremely uncommon, with only four households having tippy-taps. At critical times (e.g., before eating, before handling food, after using the latrine), most households did not report washing hands either “all of the time” or “most of the time”. One third of respondents stated that they used soap when washing hands. However, among the four households with tippy-taps, no soap was observed, and the jerry cans had not been filled with water for a long time.
Mbuya et al., 2015 [27]	Children	0–18 months	Community and Households	Zimbabwe	21 households	4 phases of formative research, comprising in-depth interviews, focus group discussions, behavior trials, and a combination of observations and microbiological sampling methods	To develop a water, sanitation, and hygiene (WASH) intervention to minimize fecal–oral transmission among children aged 0–18 months in the Sanitation Hygiene Infant Nutrition Efficacy (SHINE) trial.	Promotion of hand hygiene practices (Increased hand washing) Use and adoption of tippy-taps	Within 2 weeks of counseling, all study households had built and were using a Tippy-tap,. After 1 year, 12 of the 15 (80%) households still had a Tippy-tap installed, with evidence of use (water in the container and on the ground around the device).
Musoke et al. 2018 [28]	Household with children below 5 years Community informants Two Schools	Adults and primary school pupils (Age not provided)	Community and schools	Uganda	24 community informants 200 health club pupils 200 households	Interventions - Special training sessions on hand washing, specifically, the use of *tippy-tap* technology were conducted in the community survey and observations FGDs In-depth interviews	To improve the health status of the inhabitants through conducting community proactive and sustainable interventions targeting two priority problem areas of access to safe drinking water and improved sanitation facilities	Promotion of hand hygiene practices (Increased hand washing) Use of tippy taps	More than 200 household constructed their own tippy-taps. Use of tippy taps improved hand washing practices among adults and children especially after using the toilet.
Mwakitalima (2018) [29]	Households	Heads of households (age not provided)	Community	Tanzania	2875 households	Interviews, Observations	To evaluate the extent that the campaign has contributed to the overall coverage of improved sanitation in relation to areas that are not under the campaign?	Use and adoption of tippy-taps	About 10% (n = 252) of the households had a tippy-tap while only 3% (n = 77) households had a sink with a tap. Tippy-taps were available in 14.1% of the households in the intervention villages versus 4.1 in the control villages. Many households adopted Tippy-tap was the most adopted hand washing station compared to fised basin, mobile basin/bucked, water source such as hand pump and sink with tap
Pietropaoli (2017) [30]	Households	Adults (Age not provided)	Community	Sierra Leone	24 households	Survey? Formative research. Counselled Interventions: Construct a handwashing station (such as a tippy-tap); 3. Prioritise soap for handwashing and keeping soap by the handwashing station	Test mothers’ responses to recommendations for improving infant and young child feeding, WASH and other desired practices; and determine which practices were most feasible and acceptable Investigate the constraints mothers face when trying to change feeding patterns, hygiene practices and other daily routines and determine their motivations for trying and sustaining new practices	Use and adoption of tippy-taps	Tippy taps were accepted and used by many households. The main reasons for constructing a tippy-tap were that it was simple to construct, and that it was made from locally available materials.
Shukla (2018) [31]	15 angamwandis and 116 children	Children 2–6 years	Community	India	116 children 15 angamwandis	Quantitative: Survey -checklist for facility assessment. -tippy-tap was introduced in the anganwadis	To identify the lack of facilities in the anganwadis and implement innovative and sustainable solutions to tackle grass-root level problems at anganwadi centres	Promotion of hand hygiene practices (Increased hand washing) Use of tippy-taps	None (0%) of the children in the anganwadis practiced handwashing before the meals. Intervention was instantly accepted in the anganwadis and children started with the habit of handwash before every meals.
Singh et al. (2016) [32]	Community Health Workers (CHWs) Community Health Volunteers CHV	40 yrs. mean age (> 18 years)	Community and households	Uganda	81 CHV	In-depth Interviews with 82 CHVs. Each interview lasted from 1 to 1.5 h. These informal face-to-face interviews were semi-structured with open-ended and some Likert scale questions.	To understand whether full-time professional CHWs can potentially work with volunteers in the community to widen their reach and scope and if so what motivators might be of key importance to the CHVs remaining active in the field	Use and adoption of tippy-taps	CHVs put what they learnt into practice by building tippy-taps, having dish-racks and purifying water in their homes and acted as role models in the community. A large number of tippy-taps were built in the community. About 4.7% of households had tippy-taps at baseline compared to 47% post intervention, *P* < 0.05). The CHVs implemented what they learnt during the training and as such were role models to other members of the community with 84% of CHVs having tippy-taps themselves. This compares to 1% of CHVs who had a tippy-tap, prior to the commencement of the study.
Singh et al. (2016b) [33]	CHWs, CHV, caregivers of the underfive children	Adults (Age not provided)	Community	Uganda	4 paid supervisor (CHWs), 82 CHV 200 household	100 household intervention 100 household in control group	To compare training alone versus training and supportive supervision by paid CHWs (*n* = 4) on the effectiveness of CHVs (*n* = 82) to deliver education about pregnancy, newborn care, family planning and hygiene.	Use and adoption of tippy-taps	At 1 year follow-up there was a significantly higher prevalence of installed and functioning tippy-taps for hand washing (*p* < 0.002) in the intervention villages (47%) than control villages (35%).
Zhang et al. (2013) [34]	397 School children	7–13 years	Schools	Uganda	398 children (8 schools)	Pre−/postintervention surveys were fielded in eight schools. Four intervention schools were given tippy-taps, soap and educational materials, while four control schools initially received only educational materials. At each school, one classroom was selected at random (lottery draw), and 25 boys and 25 girls (Grades 2–5) were selected from that classroom to be given surveys using a systematic random sampling design (every third girl and boy)	To measure the efficacy of a tippy-tap-based handwashing programme in promoting handwashing rates in elementary schools in rural Uganda	Promotion of hand hygiene practices (Increased hand washing and prevention of diarrhoea)	After 1 month, the intervention schools reported a large increase in daily handwashing rates and absence of stomach pain episodes compared with the control schools. After receiving the intervention, the control schools attained similar handwashing and stomach pain rates. Both handwashing at school and after using the toilet increased after the introduction of tippy-taps. The proportion of students reporting ‘always’ or ‘often’ washing their hands at school increased from 3.5% at baseline to 100.0% at follow-up {*t* = 19.54, *P* < 0.05, 95% confidence interval (CI) 1.21–1.68 in the intervention schools [replicated in control schools by Time 3 (*t* = 12.92, *P* < 0.05, 95% CI 1.48–2.45]. The proportion of students ‘always’ washing their hands after using the toilet increased from 5.5 to 65.0% (*t* = 14.61, *P* < 0.05, 95% CI 1.02–1.58) in the intervention schools [Washing hands after using the toilet among students in the control schools increased from 3.6 to 79.3% (*t* = 13.21, *P* < 0.05, 95% CI 1.16–1.90) by Time 3]. Use of soap in the intervention schools increased from 13.5 to 84.5% (*t* = 5.64, *P* < 0.05, 95% CI 0.29–1.04) with even higher proportions reported at control schools at Time 3 (*t* = 298.15, *P* < 0.05, 95% CI 0.86–0.88). In the intervention schools, the proportion of students reporting washing their hands three or more times/day increased from 5.5 to 93.0% (*t* = 9.84, *P* < 0.05, 95% CI 0.98–1.91) after the installation of tippy-taps. Furthermore, the control schools also attained the handwashing rates of the intervention schools (97.9%) by Time 3 (*t* = 18.47, *P* < 0.05, 95% CI 1.42–2.01). Proxy data on the incidence of diarrhoeal disease are indicated by the number of students reporting stomach pain episodes in the previous month. In the intervention schools, the percentage of students reporting no stomach pain episodes increased from 7.0 to 80.0% (*t* = 10.84, *P* < 0.05, 95% CI 0.92–1.68).

### Search outcome

The search yielded a total of 4091 titles of articles of which 1696 were retained in a preliminary assessment stage after removing duplicates. Of the retained articles1623 were further excluded from the analysis because they were based on different study areas or were abstracts only. Seventy-three titles were retained, and their full articles were retrieved and assessed by two authors for eligibility. The third author validated the eligibility of the articles for inclusion in the review. From this assessment, only 20 articles met the inclusion criteria. Fifty-three articles were excluded from this systematic review because they did not meet the eligibility criteria (see Fig. 3).

### Risk of bias/quality appraisal

Quality of the design and reporting system were the main focus at this stage. Three review authors independently assessed the risk of bias in the included studies. The MMAT [35] was used to appraise the twenty studies included in the review critically. MMAT is a validated checklist used to appraise the quality of studies included in any systematic review with a quantitative, qualitative and mixed methods approach [36–38]. The MMAT has two general screening questions applicable to all study designs: 1) Are there clear qualitative and quantitative research questions or objectives, or is there a clear mixed-methods’ question or objective? 2) Do the collected data address the research question or objective? The MMAT appraises the following study methodologies and designs: qualitative, quantitative randomised controlled, quantitative non-randomized, quantitative descriptive and mixed methods study designs. The tool is divided into five components and each component is designed to assess the quality of a specific study design. These components are qualitative, quantitative randomised controlled, quantitative non-randomized, quantitative descriptive, and mixed methods studies. All components are numbered, and each section has three to four assessment criteria. For example, assessment criteria for assessing for randomised controlled trial studies included: 1) Is there a clear description of the randomization? 2) Is there a clear description of the allocation concealment? 3) Are there complete outcome data [80% or above]? 4) Is there low withdrawal/drop-out (below 20%)? Each criterion equals 25% if the assessment response is ‘Yes’, and zero if the response is ‘No’. A summation of the responses is the total score of the quality of the study in per cent and the maximum score per study is 100% (see Table 3). In the assessment component for mixed methods, 25% is given by default and is summed up with other scores from the criteria under this component. Overall, the higher the score, the better the quality of the study. MMAT was chosen to appraise studies in this review because it can simultaneously appraise studies of different designs, which suits different study methodologies included in this systematic review.

**Table 3:** MMAT

Name of study author	Type of study	Methodological quality criteria	Yes	Comments	Score
Abass (2018) [16]	Mixed methods	5.1. Is the mixed methods research design relevant to address the qualitative and quantitative research questions (or objectives), or the qualitative and quantitative aspects of the mixed methods question (or objective)?	Y	not clear	75%
		5.2. Is the integration of qualitative and quantitative data (or results*) relevant to address the research question (objective)?	Y		
		5.3. Is appropriate consideration given to the limitations associated with this integration, e.g., the divergence of qualitative and quantitative data (or results*) in a triangulation design?	N		
Aiemjoy et al. 2017 [17]	Quantitative cross sectional	4.1. Is the sampling strategy relevant to address the quantitative research question (quantitative aspect of the mixed methods question)?	Y		100%
		4.2. Is the sample representative of the population understudy?	Y		
		4.3. Are measurements appropriate (clear origin, or validity known, or standard instrument)?	Y		
		4.4. Is there an acceptable response rate (60% or above)?	Y		
Biran (2011) [7]	Qualitative	1.1. Are the sources of qualitative data (archives, documents, informants, observations) relevant to address the research question (objective)?	Y	Nothing on analysis	50%
		1.2. Is the process for analysing qualitative data relevant to address the research question (objective)?	N		
		1.3. Is appropriate consideration given to how findings relate to the context, e.g., the setting, in which the data were collected?	Y		
		1.4. Is appropriate consideration given to how findings relate to researchers’ influence, e.g., through their interactions with participants?	N		
Breese et al., (2016)	Qualitative	1.1. Are the sources of qualitative data (archives, documents, informants, observations) relevant to address the research question (objective)?	Y		100%
		1.2. Is the process for analyzing qualitative data relevant to address the research question (objective)?	Y		
		1.3. Is appropriate consideration given to how findings relate to the context, e.g., the setting, in which the data were collected?	Y		
		1.4. Is appropriate consideration given to how findings relate to researchers’ influence, e.g., through their interactions with participants?	Y		
Cantrell, (2013) [19]	Quantitative descriptive Survey	4.1. Is the sampling strategy relevant to address the quantitative research question (quantitative aspect of the mixed methods question)?	Y		100%
		4.2. Is the sample representative of the population understudy?	Y		
		4.3. Are measurements appropriate (clear origin, or validity known, or standard instrument)?	Y		
		4.4. Is there an acceptable response rate (60% or above)? applicable, an acceptable response rate (60% or above), or an acceptable	Y		
Chisanga et al. 2018 [20]	Quantitative cross sectional	4.1. Is the sampling strategy relevant to address the quantitative research question (quantitative aspect of the mixed methods question)?	Y		100%
		4.2. Is the sample representative of the population understudy?	Y		
		4.3. Are measurements appropriate (clear origin, or validity known, or standard instrument)?	Y		
		4.4. Is there an acceptable response rate (60% or above)?	Y		
Chiziwisano et al., 2019	Mixed methods	5.1. Is the mixed methods research design relevant to address the qualitative and quantitative research questions (or objectives), or the qualitative and quantitative aspects of the mixed methods question (or objective)?	Y		100%
		5.2. Is the integration of qualitative and quantitative data (or results*) relevant to address the research question (objective)?	Y		
		5.3. Is appropriate consideration given to the limitations associated with this integration, e.g., the divergence of qualitative and quantitative data (or results*) in a triangulation design?	Y		
Christensen et al. (2015) [22]	Randomized controlled trial	2.1. Is there a clear description of the randomization (or an appropriate sequence generation)?	Y		100%
		2.2. Is there a clear description of the allocation concealment (or blinding when applicable)?	Y		
		2.3. Are there complete outcome data (80% or above)?	Y		
		2.4. Is there low withdrawal/drop-out (below 20%)?	Y		
Contzen et al. (2015) [23]	Quasi-experiment	2.1. Is there a clear description of the randomization (or an appropriate sequence generation)?	Y		75%
		2.2. Is there a clear description of the allocation concealment (or blinding when applicable)?	N		
		2.3. Are there complete outcome data (80% or above)?	Y		
		2.4. Is there low withdrawal/drop-out (below 20%)?	Y		
Dajaan et al. (2018) [24]	Quantitative cross sectional	4.1. Is the sampling strategy relevant to address the quantitative research question (quantitative aspect of the mixed methods question)?	Y		100%
		4.2. Is the sample representative of the population understudy?	Y		
		4.3. Are measurements appropriate (clear origin, or validity known, or standard instrument)?	Y		
		4.4. Is there an acceptable response rate (60% or above)? applicable, an acceptable response rate (60% or above), or an acceptable	Y		
Hurtado (1994) [25]	Qualitative	1. Are the sources of qualitative data (archives, documents, informants, observations) relevant to address the research question (objective)?	Y	not clear	75%
		1.2. Is the process for analysing qualitative data relevant to address the research question (objective)?	Y		
		1.3. Is appropriate consideration given to how findings relate to the context, e.g., the setting, in which the data were collected?	Y		
		1.4. Is appropriate consideration given to how findings relate to researchers’ influence, e.g., through their interactions with participants?	N		
Kamuteera et al. 2018 [26]	Quantitative cross sectional survey	4.1. Is the sampling strategy relevant to address the quantitative research question (quantitative aspect of the mixed methods question)?	Y	not clear	75%
		4.2. Is the sample representative of the population understudy?	Y		
		4.3. Are measurements appropriate (clear origin, or validity known, or standard instrument)?	Y		
		4.4. Is there an acceptable response rate (60% or above)? applicable, an acceptable response rate (60% or above), or an acceptable	N		
Mbuya et al., (2015) [27]	Qualitative	1.1. Are the sources of qualitative data (archives, documents, informants, observations) relevant to address the research question (objective)?	Y		75%
		1.2. Is the process for analyzing qualitative data relevant to address the research question (objective)?	Y		
		1.3. Is appropriate consideration given to how findings relate to the context, e.g., the setting, in which the data were collected?	Y		
		1.4. Is appropriate consideration given to how findings relate to researchers’ influence, e.g., through their interactions with participants?	N		
Musoke et all. 2018 [28]	Mixed methods	5.1. Is the mixed methods research design relevant to address the qualitative and quantitative research questions (or objectives), or the qualitative and quantitative aspects of the mixed methods question (or objective)?	Y	Superficial analysis procedures reported	75%
		5.2. Is the integration of qualitative and quantitative data (or results*) relevant to address the research question (objective)?	Y		
		5.3. Is appropriate consideration given to the limitations associated with this integration, e.g., the divergence of qualitative and quantitative data (or results*) in a triangulation design?	N		
Mwakitalima (2018) [29]	Quantitative cross sectional	3.1. Are participants (organizations) recruited in a way that minimizes selection bias?	Y	5KM apart	100%
		3.2. Are measurements appropriate (clear origin, or validity known, or standard instrument; and absence of contamination between groups when appropriate) regarding the exposure/intervention and outcomes?	Y		
		3.3. In the groups being compared (exposed vs. non-exposed; with intervention vs. without; cases vs. controls), are the participants comparable, or do researchers take into account (control for) the difference between these groups?	Y		
		3.4. Are there complete outcome data (80% or above), and, when applicable, an acceptable response rate (60% or above), or an acceptable follow-up rate for cohort studies (depending on the duration of follow-up)?	Y		
Pietropaoli (2017) [30]	Qualitative	1.1. Are the sources of qualitative data (archives, documents, informants, observations) relevant to address the research question (objective)?	Y	Nothing on analysis	50%
		1.2. Is the process for analyzing qualitative data relevant to address the research question (objective)?	N		
		1.3. Is appropriate consideration given to how findings relate to the context, e.g., the setting, in which the data were collected?	Y		
		1.4. Is appropriate consideration given to how findings relate to researchers’ influence, e.g., through their interactions with participants?	N		
Shukla (2018) [31]	Quantitative descriptive	4.1. Is the sampling strategy relevant to address the quantitative research question (quantitative aspect of the mixed methods question)?	Y	Info not given	75%
		4.2. Is the sample representative of the population understudy?	Y		
		4.3. Are measurements appropriate (clear origin, or validity known, or standard instrument)?	Y		
		4.4. Is there an acceptable response rate (60% or above)?	N		
Singh et al. (2016) [32]	Qualitative	1.1. Are the sources of qualitative data (archives, documents, informants, observations) relevant to address the research question (objective)?	Y		100%
		1.2. Is the process for analysing qualitative data relevant to address the research question (objective)?	Y		
		1.3. Is appropriate consideration given to how findings relate to the context, e.g., the setting, in which the data were collected?	Y		
		1.4. Is appropriate consideration given to how findings relate to researchers’ influence, e.g., through their interactions with participants?	Y		
Singh et al. (2016b) [33]	Randomised controlled trial	2.1. Is there a clear description of the randomization (or an appropriate sequence generation)?	Y		75%
		2.2. Is there a clear description of the allocation concealment (or blinding when applicable)?	N		
		2.3. Are there complete outcome data (80% or above)?	Y		
		2.4. Is there low withdrawal/drop-out (below 20%)?	Y		
Zhang et al. (2013) [34]	Randomized controlled trial	2.1. Is there a clear description of the randomization (or an appropriate sequence generation)?	Y		75%
		2.2. Is there a clear description of the allocation concealment (or blinding when applicable)?	N		
		2.3. Are there complete outcome data (80% or above)?	Y		
		2.4. Is there low withdrawal/drop-out (below 20%)?	Y		

*Both qualitative and quantitative results

### Data synthesis

A narrative approach was used to synthesise data. Narrative synthesis in systematic reviews is recommended when there is a great variation in variables such as outcomes, interventions, population, and methods across studies [39]. We integrated the findings from the qualitative and quantitative findings [40]. This design involves either turning qualitative data into quantitative (quantitising) or quantitative findings are turned into qualitative (qualitising) to facilitate their integration [40]. This design has been widely used in mixed methods systematic reviews [41, 42]. We used study outcomes as themes to synthesise data. A narrative approach was also used to synthesis the quality of study and characteristics of the study characteristics.. The main category of the analysis was based on the promotion of handwashing behaviour by using tippy-tap. Under this category, the reviewers came up with three subcategories, namely: the use and benefit of tippy-tap in promoting hand hygiene; adoption of tippy-tap and its associated hand hygiene resources, and the effectiveness of tippy-tap. In this systematic review, “use” of tippy-tap refers to the situation whereby the participant merely used tippy-tap to wash hands and/or increased their handwashing during their respective project implementation. On the other hand, “adoption” of tippy-tap refers to a situation whereby the participant continued using tippy-taps even after their respective research projects or programs had stopped or constructed new tippy-taps after completion of the project. Effectiveness of tippy-tap in this study refers to proxy data of reducing infectious diseases. Content analysis was carried out to synthesise the extracted data and similar information was grouped (see Table 2). Findings were presented in narrative form as shown below. The interventions were also classified according the settings where they were implemented. The settings of the study were classified as households (peoples’ houses), primary schools, and communities. Community based intervention in this study refers to interventions implemented at a public place (village level, church, and neighbourhoods). Statistical meta-analysis was not possible as the studies varied considerably on how the study outcomes were analysed by the researchers.

## Results

### Quality appraisal

Based on MMAT, nine studies scored 100% [17–22, 24, 29, 32]. Of these, two were qualitative, five were quantitative descriptive, and one was a mixed-methods study. Nine studies scored 75% [16, 23, 25–28, 31, 33, 34] among these, three were experimental studies that had no information on blinding [23, 33, 34]; three were qualitative studies with no clear description regarding the influence of the researcher on study findings [25, 27, 31]; two were mixed methods studies that did not highlight the limitations to integration of qualitative and quantitative findings [16, 28]; and one was a non-randomised study with a low response rate [26]. Two qualitative studies scored 50% each because they lacked information about how data were analysed and description on whether a special consideration was given to how findings related to the researcher’s influence [7, 30]. MMAT has no cut-off point for the quality of studies, but we considered ‘less than 50%’ score as low quality. However, none of our selected studies scored below 50%. With an average MMAT score of 82.5% across the included studies, the studies are considered to be of high quality.

### Study characteristics

Twenty studies met the eligible criteria. Of these, six were conducted in Uganda [7, 26, 28, 32–34], two in Ethiopia [17, 23], and two in Tanzania [20, 29]. Furthermore, one study was conducted in each of the following countries: Zambia [18], Zimbabwe [27], Kenya [22], Nigeria [16], Haiti [19], Malawi [21], Ghana [24] and Sierra Leone ([30] (See Table 2). In terms of study design, six qualitative studies [7, 18, 25, 27, 30, 32], 11 quantitative studies [17, 19, 20, 22–24, 26, 29, 31, 32, 34], and three mixed methods studies [16, 21, 28] were evaluated. Data collection in the qualitative studies was through focus group interviews, semi-structured questionnaire and in-depth interviews. The quantitative studies utilised quasi-experiment, pre-post survey, cross-sectional survey and cluster randomised trials study approaches (see Table 2).

A total of 11 studies were conducted in the community [7, 16, 17, 19–23, 25–30] and four were conducted in schools [18, 24, 28, 34]. The study population in six studies were children while 16 studies were conducted with adults. The youngest participants were infants less than 8 months old [27] and the oldest was 40 years [32] The number of participants in each study varied from 21 [18] to 2875 [29].

### Summary of the findings

Studies included in this review were analysed based on the following three outcomes: the use and benefit of tippy-tap in promoting hand hygiene; adoption of tippy-tap and its associated hand hygiene resources, and the effectiveness of tippy-tap. These sub-categories were generated from the objective of the study. The presentation and interpretation of the results follow these categories as narrated below.

### Use and benefits of tippy-tap in promoting hand hygiene

The use of tippy-taps for handwashing among household members or school children was reported by authors of 16 studies conducted in Nigeria, Haiti, Malawi, Ghana, India, Tanzania, Uganda, Sierra Leone, Kenya and Ethiopia [7, 16, 17, 19–26, 28–31, 34]. The use of tippy-tap among the participants in the 16 studies ranged from 2.7% [26] to 80% [20].

Concerning the benefits of using tippy-taps, authors of three studies [7, 23, 34] reported an increase in handwashing practice by participants after being exposed to tippy-tap. In a randomised controlled trial in Uganda four intervention and four control schools were recruited into the study [34]. At each school, one classroom was selected randomly (lottery draw), and 25 boys and 25 girls (Grades 2–5) were selected from that classroom using a systematic random sampling design (every third girl and boy). Data were collected at three waves of 1 month apart intervals. The first wave was a baseline survey that was followed by the provision of soap and handwashing education to four intervention schools. The second wave was followed by the introduction of tippy-taps and provision of soap to the intervention group. Lastly, the post-intervention survey was carried out at the last wave. The four control schools received health education only through-out the experiment and were provided with tippy-taps post-study interventions. The researchers reported an increased estimate in the proportion of students reporting ‘always’ or ‘often’ washing their hands at school from 3.5% at baseline to 100.0% at follow-up (*t* = 19.54, *P* < 0.05, 95% CI 1.21–1.68) in the intervention schools. When the similar intervention was replicated in the control schools by Time 3, there was an increase in handwashing (*t* = 12.92, *P* < 0.05, 95% CI 1.48–2.45] [34]. In the same study, it was observed that the proportion of students ‘always’ washing their hands after using the toilet increased from 5.5 to 65.0% (*t* = 14.61, *P* < 0.05, 95% CI 1.02–1.58) in the intervention schools, while in the control schools it only increased from 3.6 to 79.3% (*t* = 13.21, *P* < 0.05, 95% CI 1.16–1.90) by Time 3 when the same intervention was replicated [34].

In addition, compared to control schools, introduction of tippy-taps increased the use of soap by students in the intervention schools in an experiential study from 13.5 to 84.5% (*t* = 5.64, *P* < 0.05, 95% CI 0.29–1.04); handwashing from 5.5 to 93.0% (*t* = 9.84, *P* < 0.05, 95% CI 0.98–1.91) and handwashing after using the toilet from 5.5 to 65.0% (*t* = 14.61, *P* < 0.05, 95% CI 1.02–1.58) [34]. Similarly, another study [7] found that tippy-taps increased handwashing after latrine use by providing convenient soap and water, and by acting as a salient cue to handwashing. Although quantitative data on handwashing rates were not collected, participants in households with tippy-taps believed that their post-latrine handwashing rates had increased as a result of the tippy-taps [7]. Pre- and post-data analysis on self-reported handwashing revealed that the population-tailored interventions, especially the tippy-tap-promotion, performed better than the standard education intervention (education intervention, the f-diagram exercise, an often applied intervention tool) [18]. In a study conducted by Christensen and colleagues [22], the use of tippy-tap was measured through the availability of handwashing resources (soap and water) at the tippy-tap station. These researchers found that enumerator-observed indicators of use were still high (72–85% for having both soap and water present at the tippy-tap station) [22]. In an Indian qualitative study, most participants reported using tippy-tap because of its benefits [25]. The participants reported that handwashing using tippy-tap requires less water and soap compared to the usual method of handwashing [25]. However, in the same study [25] participants indicated the following as challenges of the tippy-tap handwashing technology: it was not easy to wash hands of very young children with the tippy-tap; there was a potential problem that older children may play with the device, thus destroying it or wasting water; it was also recognized that the device required extra water, time, and work to install, use, and maintain. In addition, a study by Biran [7], one participant suggested that tippy-taps did not look attractive, elderly participants said tippy-taps looked childish and unnecessary, and that people used to live longer even before tippy-taps were developed.

On the other hand, the economic benefits of tippy-taps were reported by the authors of a Nigerian study [16]. The installation of tippy-taps in small scale business facilities by women who were involved in selling food items led to an increase in the number of customers, which resulted in more sales and profits.

### Adoption of tippy-tap and its associated hand hygiene resources

Authors of six studies assessed the adoption of tippy-taps by households [7, 18, 22, 23, 32, 33]. In a study conducted by Christensen [22], the intervention households were significantly more likely to have a place for handwashing (71–85 percentage point increases) with soap available (49–66 percentage point increases) than controls. These authors also noted an increase of 86% in having a dedicated location for tippy-taps. Similarly, in another study, teachers educated school going children on tippy-tap as a handwashing station [18]. Although these children were not directly asked to construct tippy-tap, they all managed to attempt building one or influence their parents to assist them. Their parents trusted the information received from their children. The tippy-taps were also found to be attractive, easy to use and helpful in fostering the habit of handwashing among children [18].

Signh et al. [33] engaged the community in a hand hygiene promotion program. At 1 year follow-up, the researchers noted a 47% installation of functioning tippy-taps in the intervention villages compared to 35% in the control villages (p < 0.002) [33]. There was a significant increase in tippy-tap installation by community members from 4.7% of households at baseline to 47% of homes after the intervention, following the demonstrations to construct the device by community health volunteers (CHVs). The CHVs were trained on the tippy tap construction and acted as role models to other community members. Furthermore, there was a great improvement in owning tippy-taps by CHVs from 1% at baseline to 84% after interventions [33]. Another significant evidence of adoption of tippy-taps was observed in a study where all study households built tippy-taps within 2 weeks of counselling [27]. After 1 year of tippy-tap promotion, 80% of the households still had a tippy-tap installed, with evidence of use (water in the container and on the ground around the device). Similar results were observed in a study by Contzen and colleagues [23] in which, close to 100% of the households followed the promotion and invested material and time to construct their tippy-tap. In the same study, all participants in the intervention group constructed tippy-taps and about 83% of these were still operational 3 months after termination of the interventions.

Although there is limited awareness on tippy-tap, having knowledge about tippy-tap did not result in immediate construction of the station [7, 18]. The researcher thought that study participants constructed a tippy-taps because they were asked to do so, or they anticipated that the researcher would be visiting them regularly to evaluate the adoption of the technology [7]. Some participants constructed tippy-tap as a result of campaigns and fear of fines from community leaders [7].

### Effectiveness of tippy-tap

Out of twenty articles under review, only one study [34] had an incidence of diarrhoea as an outcome measure. The study was conducted in a school setting in Uganda and aimed at measuring the efficacy of a tippy-tap-based handwashing programme in promoting handwashing rates in elementary schools in rural Uganda. Zhang and colleagues [34] used the pre-and post-intervention surveys in which four intervention schools were given tippy-taps, soap and educational materials, while four control schools initially received only educational materials. Proxy data for assessing the effectiveness of tippy-taps in reducing diarrhoeal disease was indicated by the number of students reporting stomach pain episodes in the previous month. The authors of the study found that in the intervention schools, the percentage of students reporting no stomach pain episodes increased from 7% at baseline to 80% after the intervention (*t* = 10.84, *P* < 0.05, 95% CI 0.92–1.68) [34]. However, no proxy data was provided on the trend of diarrhoea in the control group.

## Discussion

The aim of this systematic review was to assess the use, benefits, adoption and effectiveness of tippy-tap handwashing station in resource-limited settings. A total of twenty articles were identified and reviewed. The findings of our systematic review show that the availability of tippy-taps increased handwashing and use of soap among participants. Furthermore, the majority of people who were oriented to tippy-taps or recruited to tippy-tap studies built their tippy-tap stations even after the end of promotional activities or programs. In one study, tippy-taps were found to be effective in preventing stomach pain episodes among participants [34].

There is sufficient evidence that hand washing is a single most important intervention for preventing diarrhoeal and respiratory infections, yet the rate of handwashing in resource-limited settings is very low [3, 43–45]. Indeed, with frequent global outbreaks of infectious diseases such as COVID-19, and Ebola, the importance of identifying a cost-effective hand handwashing enabling technologies cannot be overemphasized. The findings of this review suggest that tippy-taps have a great potential to improve the health outcomes of people as it increases handwashing and use of soap, which are crucial in breaking the transmission cycle of infections. The findings of our study point to many advantages of tippy-taps over other hand washing station technologies. These advantages include inexpensive to construct as it uses local materials, easy to construct, entertaining for children, water economical and convenient to use as it is usually constructed near the toilet so that people can easily wash their hands after using the toilet.

In addition, the findings of this study indicate that tippy-taps have a higher likelihood of being adopted by participants. Our study findings show that the majority of participants who constructed their tippy-taps were still using them even after the end of interventions or promotional programs [7, 23, 27, 32, 34]. This is not surprising given that tippy-taps are cost-effective and are made from locally available resources [6, 46]. Evidence points to the following as facilitators of adoption of public health interventions by users: perceived importance of the intervention, availability of resources, affordability, culturally appropriate, easy to use, availability of technical and financial support [47].

Furthermore, although more than three decades have passed since the first tippy-tap was constructed, the findings of our systematic review demonstrate that there is still limited data regarding its use and effectiveness. Only a few studies have specifically evaluated tippy-tap as an intervention. Out of the 20 studies included in this study, only three were experimental studies [22, 23, 34]. Out of these three experimental studies, only one [34] specifically evaluated the effectiveness of the tippy-tap in preventing stomach pain episodes. In the other two studies [22, 23], tippy-taps were part of a combined water and hygiene interventions that were evaluated together. While Zhang and colleagues [34] reported that tippy-taps were effective in reducing stomach pains episodes among the participants in the treatment group, the study lacked information regarding blinding of participants and measures of fidelity which put to question the validity and reliability of the findings.

The findings of this review suggest that there is a dearth of literature on tippy-tap enabling technology especially on the promotion of handwashing practices. The history of tippy-tap dates to 1980s, but the first peer-reviewed article was published in 1994 [25] . Thereafter, a gap ensued until 2011 when Biran and colleagues published the next paper on tippy-tap [7]. Our search strategy indicated that the latest articles in this field were published in 2019 [21, 31] while the remaining studies were conducted between 2011 and 2019.

### Limitations of the study

This review is not without limitations. First, our systematic review only included studies that were conducted in English. This may have introduced bias to the findings of the study as some studies published in other languages may have had information that could be useful in answering the research question. Second, the study was limited to poor resource countries limiting the generalisation of the findings to other settings. Notwithstanding these limitations, all the studies except two, scored high (≥75%) on quality appraisal using MMAT with eight articles scoring 100%. This entails that the majority of the studies included in this review were of moderate or strong quality.

### Implications of the study findings for practice, research and policy

The findings of this systematic review of literature inform practitioners, policy makers and researchers about the use, adoption, benefits, and effectiveness of tippy-taps in resource limited countries. The tippy-tap technology is one of the interventions that people working in the field should promote in resource-limited settings where the majority of people fetch water from community boreholes or wells which are far from their houses. Tippy-taps are cheap, easy to construct, entertaining to children, and easy to adopt which make them suitable hand washing promotion intervention in resource poor countries where the prevalence of waterborne and other infectious diseases is high. Public health care workers, Governments, non-governmental organisations, and other stakeholders are encouraged to take a leading role in promoting the use of tippy-taps to people through public campaigns. The campaigns may target schools, churches, communities, and hospitals where majority of the people can be reached. In addition, use of mass media such as radio and television could also be used to educate people about the importance of tippy-taps. Trainings for community volunteers are also needed to empower them with information on how they can support communities to build their own tippy-taps. Another important issue to consider is that we only identified one study that evaluated the effectiveness of the tippy-taps. Moreover, the study had some methodological problems that impacted on the validity and reliability of the findings. Thus, evidence on the effectiveness of tippy-taps in preventing infectious disease is still limited. Rigorous interventional studies with fidelity measures are needed to evaluate the effectiveness of tippy-taps in reducing waterborne and other infectious diseases. Furthermore, although schools are places where children spend much of their time, interact with others and easily get or transmit infections, only five studies [18, 24, 28, 34] included in this review had schools as a study setting. Future studies conducted in school settings are therefore necessary. Government policies that can promote the use of tippy-taps by providing subsidies or empowering communities and households through trainings to build and use tippy-taps are also needed (Hayes et al. 2019).

## Conclusion

Tippy-taps have great potential to improve health outcomes of people living in resource-limited settings where waterborne diseases are common. However, with limited data, it is difficult to ascertain how common tippy-taps are within the community or how effective they are in reducing infections associated with poor hand hygiene. More prevalence and experimental studies are warranted to provide a good understanding of the use, adoption, and effectiveness of tippy-taps. To the best of the authors’ knowledge, this is the first systematic review to assess the use, adoption, and effectiveness of tippy-tap handwashing station in promoting hand hygiene practices in a resource-limited setting.

## Supplementary information

**Additional file 1:** Supplementary material A. Standardised data extraction form

## Data Availability

The data and materials used in this systematic review are available from the corresponding author on request.

## Abbreviations

AMED: AMED Allied and Complementary Medicine
CINAHL: Cumulative Index of Nursing and Allied Health Literature
COCHRANE: Cochrane database of systematic reviews
COVID-19: Coronavirus disease 19
DOAJ: The Directory of Open Access Journals
EMBASE: Excerpta Medica dataBASE
MMAT: The Mixed Methods Appraisal Tool
PRISMA: Preferred Reporting Items for Systematic Reviews and Meta-Analyses
PROSPERO: The International Prospective Register of Systematic Reviews
PsycINFO: Psychological Information Database
UNICEF: The United Nations International Children’s Emergency Fund
UNC: University of North Caroline
WHO: World Health Organisation
