# Supplementary material for: Use, adoption, and effectiveness of tippy-tap handwashing station in promoting hand hygiene practices in resource-limited settings: a systematic review
Source: BMC Public Health. 2020 Jun 26;20:1005. doi: 10.1186/s12889-020-09101-w (PMC7316639; doi:10.1186/s12889-020-09101-w)
Supplement: Supplementary file 1 — Additional file 1: Supplementary material A. Standardised data extraction form [file 12889_2020_9101_MOESM1_ESM.docx]

**Supplementary material A: Standardised data extraction form:**

| Reviewer’s Name: |  |
| --- | --- |
| Date data extraction completed: |  |
| Study ID number: |  |
| **Citation Details** |  |
| Authors: |  |
| Title: |  |
| Journal: |  |
| Year: |  |
| Issue: |  |
| Volume: |  |
| Pages: |  |
| **Study Details** |  |
| Study design: |  |
| Country: |  |
| Setting/Context: |  |
| Type of participants: |  |
| Age of participants: |  |
| Sample size: |  |
| Method and study design: |  |
| Study aim/s: |  |
| Study objectives: |  |
| Intervention description: |  |
| Follow up duration: |  |
| Phenomena of interest |  |
| Study outcomes: |  |
| Study measures: |  |
| Year/ timeframe for data collection: |  |
| Recruitment eligibility criteria: |  |
| Method of data analysis: |  |
| Description of main results: |  |
| Ethics approval: |  |
| Authors conclusion: |  |
| **Reviewer’s comment:** |  |
